# Before and after the earthquake: a case study of attrition from the HIV antiretroviral therapy program in Haiti

**DOI:** 10.3402/gha.v7.24572

**Published:** 2014-08-05

**Authors:** Nancy H. Puttkammer, Steven B. Zeliadt, Jean Gabriel Balan, Janet G. Baseman, Rodney Destiné, Jean Wysler Domerçant, Jean Marie Duvilaire, Nernst Atwood Raphael, Kenneth Sherr, Krista Yuhas, Scott Barnhart

**Affiliations:** 1 International Training and Education Center for Health (I-TECH), Department of Global Health, University of Washington, Seattle, WA, USA; 2 Department of Health Services, University of Washington, Seattle, WA, USA; 3 International Training and Education Center for Health (I-TECH) Haiti Program, Pétionville, Haiti; 4 Division of Global HIV/AIDS, Centers for Disease Control and Prevention, Port-au-Prince, Haiti; 5 Hôpital St. Antoine de Jérémie, Ministère de la Santé Publique et de la Population d'Haïti, Jérémie, Haiti; 6 Department of Global Health, University of Washington, Seattle, WA, USA; 7 Center for AIDS Research Biometrics Core, University of Washington, Seattle, WA, USA

**Keywords:** HIV/AIDS, antiretroviral therapy, attrition, Haiti, earthquake

## Abstract

**Background:**

On January 12, 2010, a devastating 7.0 magnitude earthquake struck the West Department of Haiti, killing more than 200,000 people and injuring or displacing many more. This disaster threatened continuity of HIV care and treatment services.

**Objectives:**

This case study examined the effect of the devastating 2010 earthquake in Haiti on attrition from the HIV antiretroviral therapy (ART) program.

**Design:**

The study triangulated retrospective data from existing sources, including: 1) individual-level longitudinal patient data from an electronic medical record for ART patients at two large public sector departmental hospitals differently affected by the earthquake; and 2) aggregate data on the volume of HIV-related services delivered at the two hospitals before and after the earthquake.

**Methods:**

The study compared ART attrition and service delivery in Jacmel, a site in the ‘very strong’ zone of earthquake impact, and in Jérémie, a site in the ‘light’ zone of earthquake impact. The analysis used time-to-event analysis methods for the individual-level patient data, and descriptive statistical methods for the aggregate service delivery data.

**Results:**

Adjusted ART attrition risk was lower at the hospital in Jacmel after vs. before the earthquake (HR=0.51; *p*=0.03), and was lower in Jacmel vs. Jérémie both before (HR=0.55; *p*=0.01) and after the earthquake (HR=0.35; *p*=0.001). The number of new ART patient enrollments, new HIV patient registrations, and HIV clinical visits dropped notably in Jacmel immediately after the earthquake, but then rapidly rebounded. On average, there was no change in new ART enrollments per month after vs. before the earthquake at either site.

**Conclusion:**

These findings underscore the resilience of Haitian ART providers and patients, and contribute evidence that it is possible to maintain continuity of ART services even in the context of a complex humanitarian crisis.

On January 12, 2010, a devastating 7.0 magnitude earthquake struck the West Department of Haiti, within 30 km of the capital, Port-au-Prince. Government officials estimated that 220,000–250,000 people were killed (1). Approximately 300,000 people were injured, 300,000 homes were damaged or destroyed, and 1.3 million people were displaced (1, 2). In the 3 weeks after the earthquake, approximately 750,000 people moved out of and into Port-au-Prince, reflecting a tremendous level of disruption in Haitian society during the post-earthquake period (3).

HIV/AIDS is an important public health problem in Haiti, and the earthquake threatened to undercut many of Haiti's successes in HIV prevention, care and treatment (4). With a national HIV prevalence rate of 2.2% among adults (5), Haiti is home to more than half of all people living with HIV in the Caribbean region (6). Prior to the earthquake, Haiti had 26,000 active patients on HIV antiretroviral therapy (ART), representing approximately 68% of those meeting eligibility criteria for ART (7). Retention of patients within the national ART program had been a concern before the earthquake, with death or loss to follow-up of approximately 35% of patients ever enrolled on ART (7), and this concern escalated following the earthquake. As has been noted in natural disasters in other settings, patients with chronic disease can experience additional health challenges due to secondary physical effects of trauma, new mental health issues including acute stress, depression, and post-traumatic stress disorder, as well as interruption in self-management strategies as they deal with personal losses and disruptions in customary health services (2). In the immediate aftermath of Haiti's earthquake, there was anecdotal evidence of HIV patients who interrupted ART after losing their personal medication supplies or their connections to health facilities with records about their medication regimens (8). ART patients living in displaced persons camps faced lack of privacy, stigma, transportation challenges, and other notable stressors which compromised their continuity of care (9).

The earthquake compounded other public health challenges in Haiti. A household survey carried out in Port-au-Prince immediately before and after the earthquake estimated that 55% of households, representing approximately 1.4 million people, experienced food insecurity with moderate or severe hunger during the 6 weeks following the earthquake (1). The settlement of thousands of residents in internally displaced person camps and the disruption to already-inadequate water and sanitation facilities created a fertile environment for infectious disease. In October, 2010, a cholera outbreak claimed its first victims. By the end of 2010, cholera had spread to every Department of Haiti and more than 150,000 cases and 3,500 deaths were reported (10). These new public health challenges placed vulnerable patients, including those living with HIV, at elevated risk of poor health outcomes.

## Present investigation

### Background

This case study examines the effects of Haiti's earthquake on the phenomenon of ART attrition, or the drop-out of patients from the national ART program due to death or other reasons for loss-to-follow-up. Specifically, the case study examined data from two large public-sector departmental hospitals, one which experienced profound disruption as a result of the earthquake and the other which did not. The study explored differences in characteristics of new ART patients, differences in ART attrition risk after adjustment for patient characteristics, and differences in HIV service delivery patterns by site among patients newly enrolled on ART before vs. after the earthquake.

Our case study adds to the existing base of evidence on the effects of Haiti's earthquake on continuity of ART services. A prior study by Walldorf et al. (11) found that the aggregate number of current ART patients dropped by approximately 10% in areas intensely affected by the earthquake in the 3 months after the earthquake, but rebounded to pre-earthquake levels by 5 months after the earthquake (11). Our study complements the Walldorf et al. study by considering the time period up to 18 months after the earthquake and by adjusting for individual-level characteristics of patients enrolled on ART before vs. after the earthquake. By exploring the hypothesis that HIV-related services were negatively impacted in the area of greater earthquake intensity, this case study contributes to the literature on the relationship between natural disaster and continuity of ART services.

## Methods

### Study setting and patient population

The study cohorts included patients who initiated ART at Hôpital St. Michel in Jacmel and Hôpital St. Antoine in Jérémie from July 12, 2008 to January 11, 2010 and January 12, 2010 – July 11, 2011, periods representing 18 months before and after the earthquake. Both sites are the leading secondary hospitals in their largely rural Departments, serving catchment populations of approximately 575,000 (South East Department) and 425,000 (Grand Anse Department) respectively (12).

The sites were differentially affected by the earthquake. The earthquake epicenter was<30 km from Jacmel, and the hospital and its surroundings experienced ‘very strong’ earthquake impact, according to the Modified Mercalli Intensity Scale (MMI), as measured by the US Geological Service (13). The mayor of Jacmel estimated that 300–500 city residents were killed and another 4,000 were seriously injured (14), representing approximately 1 and 10% of the population, respectively (12). Another 4,500 residents were left homeless (15). Several buildings on Hôpital St. Michel's campus were badly damaged in the earthquake, and for weeks afterwards care was provided in the open-air courtyard under tarps.

In contrast, Hôpital St. Antoine in Jérémie is located >150 km from the earthquake epicenter, and is in the zone with direct effects of the earthquake falling in the ‘light’ category of the MMI (13). Still, Jérémie experienced some degree of upheaval as a result of the earthquake. Approximately 10,000 people from Port-au-Prince relocated to the commune of Jérémie within 3 weeks after the earthquake (3), swelling the population within the commune by about 8% (12). In theory, at Hôpital St. Antoine, services could have been disrupted as existing patients or health care workers traveled to help injured friends and relatives, as newly injured patients from the earthquake zone arrived in Jérémie seeking care, or as health system resources were diverted to sites damaged in the quake. However, it can be hypothesized that these disruptions would have been less severe than those in Jacmel.

### Data sources

Data for the case study come from the iSanté data system, the Haitian Ministry of Public Health and Population's (MSPP) electronic medical record system (16–18). The study cohort included adult patients (≥15 years) placed on a life-long ART regimen (as opposed to a short-course regimen for HIV prophylaxis). Aggregated data on counts of patients enrolling in HIV care and initiating ART at the two hospitals came from the iSanté data system and represent unduplicated patient counts. Aggregate data on the number of HIV clinical visits occurring at each site also came from iSanté but do not represent unduplicated patient counts.

### Outcome variables

**Fig. 1:**
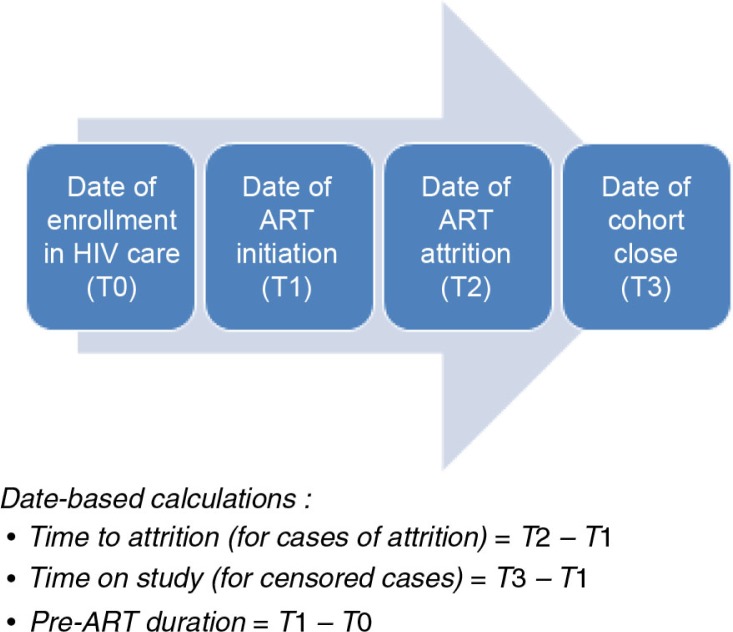
Steps in HIV care process.

The outcome variable for the time-to-event analysis at the two hospitals was time in days from ART initiation to attrition, defined as the first incident of failure to return for pharmacy pick-up within 30 days of the expected ART refill date (19). While patients typically are prescribed 30 days of ART medication at each visit, the actual amount of medication dispensed ranged above and below this value; the refill date was calculated based on the amount of medication given to the patient at the prior visit. Among patients enrolled on ART before the earthquake, the median amount of medication dispensed was for 35 days (interquartile range: 22–40 days) while among patients enrolled on ART after the earthquake the median was for 33 days (interquartile range: 30–43 days). Patients with evidence of ART attrition were considered to be censored as of January 11, 2010 for the before-earthquake cohort, or July 11, 2012 for the after-earthquake cohort. Figure 1 illustrates the trajectory of significant events for patients included in the study and how time to attrition and time to censoring were calculated. In sensitivity analysis, we used a patient deduplication algorithm in iSanté to examine whether patient transfers to other facilities explained some of the cases of attrition.

### Covariates

**Fig. 2:**
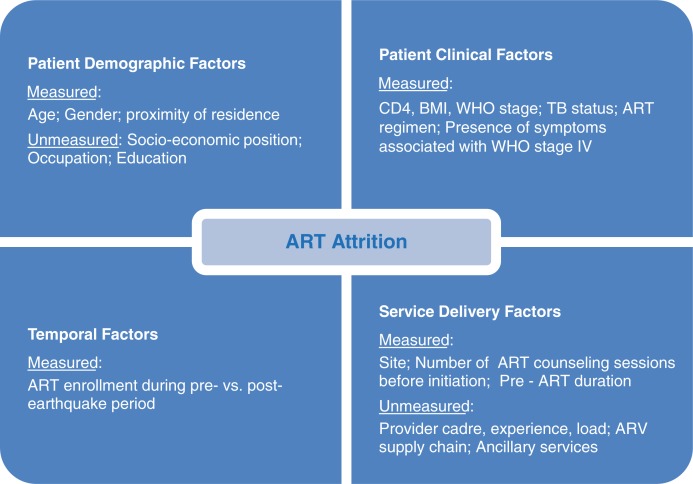
Conceptual model for factors associated with ART attrition.

Covariates for the time-to-event analysis at the two hospitals are represented in the conceptual model in Fig. 2, showing factors which explain the phenomenon of ART attrition. These included timing of ART initiation before vs. after the earthquake, age at ART initiation, gender, patient location of residence, starting ART regimen, body mass index (BMI), CD4 T-cell count, level of HIV disease progression according to the World Health Organization's staging criteria (WHO stage), presence of symptoms consistent with WHO stage IV, tuberculosis (TB) status, duration of enrollment in HIV care before ART initiation (called ‘pre-ART duration’), and number of counseling sessions before ART initiation (19). All clinical factors were measured during a baseline period 180 days prior to ART initiation. The covariates for baseline BMI and CD4 count were grouped into clinically-meaningful categories which also avoided sparse counts in any category. In sensitivity analysis, we considered other empirical ways of categorizing these covariates.

### Statistical methods

For the case study, the Chi-square test of proportions, based on categorical versions of all covariates, was used to compare characteristics of patients enrolling on ART before vs. after the earthquake. Average rates of attrition per 100 person years both overall and by patient sub-groups were estimated. The numerator for the rate was number of cases of attrition, while the denominator was the total of time to attrition plus time on study (see Fig. 1), expressed in 100 person years. The Kaplan-Meier method was used to estimate the time-to-event function for ART enrollees, and the Peto-Prentice test, a method sensitive to detection of early differences, was used to test for equality of the time-to-event function before and after the earthquake (20). A semi-parametric Cox regression model was used to explore the relationship among hospital site, timing of ART initiation before vs. after the earthquake, and attrition risk after adjustment for patient characteristics (20). The adjusted model included all covariates, a temporal variable for pre- vs. post-earthquake enrollment on ART, as well as an interaction term to detect whether the temporal effect varied by hospital site. We used multiple imputation using chained equations (21, 22) to handle missing data for the following covariates: location of patient residence relative to the hospital (0.3% missing), WHO stage (2.1% missing), BMI (8.9% missing), and CD4 level (12.9% missing). We compared trends in the number of new ART enrollments, new HIV patient registrations, and HIV clinical visits per month using autoregressive integrated moving-average (ARIMA) models which account for serial correlation of time series data. In analyses stratified by site, we tested whether there was evidence that the monthly average changed after the earthquake. All analyses were performed using STATA 12.0 statistical software (StataCorp, College Station, TX).

### Ethical review

The study used existing, de-identified patient data from the iSanté data system. The study protocol was reviewed and approved by the University of Washington, the Haiti National Bioethics Committee, and the US Centers for Disease Control and Prevention.

## Results

There were 1,012 patients in the time-to-event analysis newly enrolled on ART at the two hospitals, with 545 enrolled before the earthquake and 467 enrolled after. Patient characteristics are described in Table 1. Patients who initiated ART after the earthquake were somewhat less likely to reside in the same commune as the hospital (*p*<0.10 for the Chi-square test of heterogeneity across covariate categories), a result which was driven by differences over time in Jérémie. They were also somewhat less likely to have a baseline CD4 value of <100 cells/µl (*p*<0.10 for the Chi-square test of heterogeneity across covariate categories), a result driven by differences over time in Jacmel. They were more likely to use the ART regimen of zidovudine–lamivudine–efavirenz (AZT-3TC-EFV) as their starting regimen and to have a WHO stage IV symptom noted prior to ART initiation (*p*<0.01 for the Chi-square test of heterogeneity across covariate categories), results driven by similar differences over time at both sites. Finally, they were less likely to have no counseling sessions prior to ART initiation (*p*<0.01 for the Chi-square test of heterogeneity across covariate categories). Interestingly, the pattern on pre-ART counseling diverged by site; in Jacmel the proportion of patients with no counseling sessions fell from 52.7% before to 47.3% after the earthquake (*p*<0.001) while in Jérémie, the proportion climbed from 40.4% before to 57.2% after the earthquake (*p*<0.001).

**Table 1:** Patient characteristics before and after 2010 earthquake at two hospitals in Haiti

	Overall		Before		After	
	
	*N*	%	*N*	%	*N*	%
All patients	1,012	100.0	545	53.9	467	46.1
Site						
Jérémie	534	52.8	299	54.9	235	50.3
Jacmel	478	47.2	246	45.1	232	49.7
Gender						
Male	417	41.2	224	41.1	193	41.3
Female (non-pregnant)	529	52.3	287	52.7	242	51.8
Female (pregnant)	66	6.5	34	6.2	32	6.9
Age group						
<25	99	9.8	55	10.1	44	9.4
25–44	629	62.2	334	61.3	295	63.2
≥45	284	28.1	156	28.6	128	27.4
Commune of residence^a^						
Same	594	58.7	330	60.6	264	56.5
Adjacent	235	23.2	132	24.2	103	22.1
Non-adjacent	180	17.8	81	14.9	99	21.2
Missing	3	0.3	2	0.4	1	0.2
BMI						
<18.5	280	27.7	161	29.5	119	25.5
18.5+	642	63.4	338	62.0	304	65.1
Missing	90	8.9	46	8.4	44	9.4
Baseline CD4^a^						
<100	208	20.6	127	23.3	81	17.3
100–249	361	35.7	195	35.8	166	35.6
250+	312	30.8	157	28.8	155	33.2
Missing	131	12.9	66	12.1	65	13.9
ART regimen^b^						
AZT-3TC-EFV	542	53.6	264	48.4	278	59.5
AZT-3TC-NVP	376	37.2	223	40.9	153	32.8
Other	94	9.3	58	10.6	36	7.7
WHO stage						
Stage I or II	644	63.6	352	64.6	292	62.5
Stage III or IV	347	34.3	181	33.2	166	35.6
Missing	21	2.1	12	2.2	9	1.9
Any stage IV symptom^c^						
No	803	79.4	456	83.7	347	74.3
Yes	209	20.7	89	16.3	120	25.7
TB (treatment, prophylaxis, suspicion)						
No	882	87.2	480	88.1	402	86.1
Yes	130	12.9	65	11.9	65	13.9
Pre-ART duration						
<1 month	390	38.5	213	39.1	177	37.9
1–6 months	310	30.6	158	29.0	152	32.6
>6 months	312	30.8	174	31.9	138	29.6
Counseling sessions^c^						
None	585	57.8	322	59.1	263	56.3
1	340	33.6	193	35.4	147	31.5
2+	87	8.6	30	5.5	57	12.2

ART, antiretroviral therapy; AZT, zidovudine; BMI, body mass index; EFV, efavirenz; NVP, nevirapine; TB, tuberculosis; WHO, World Health Organization; 3TC, lamivudine.

a *p*≤0.10,

b *p*≤0.01,

c *p*≤0.001.

**Fig. 3:**
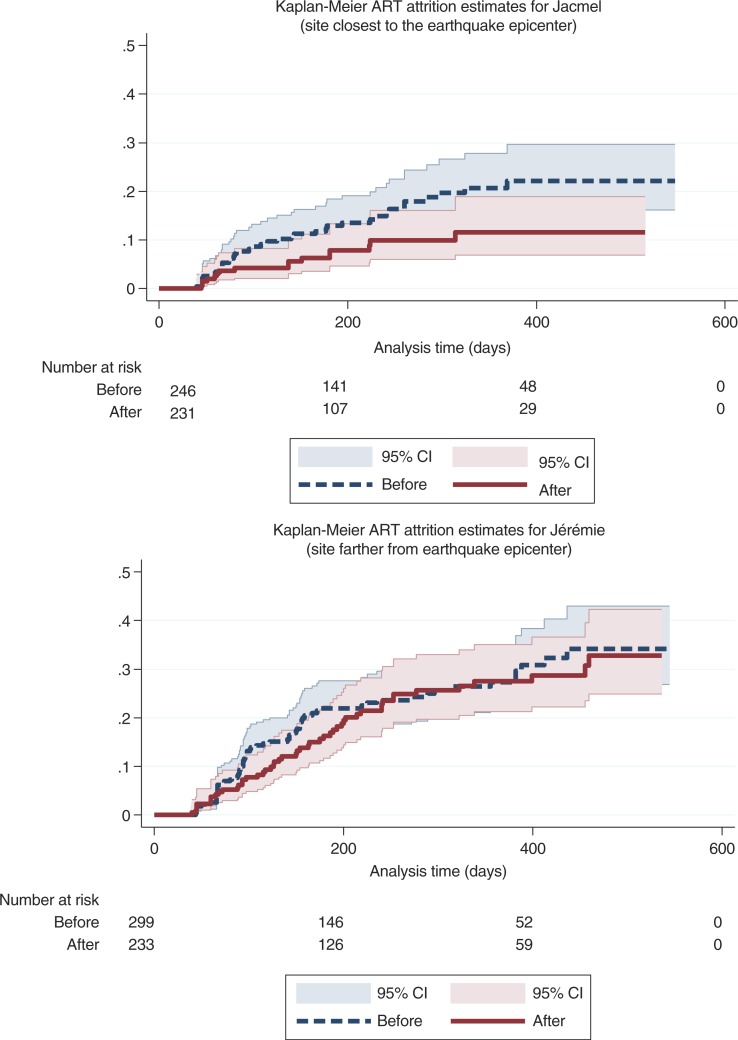
ART attrition, before and after 2010 earthquake at two hospitals in Haiti.

A total of 172 (17.0%) patients at the two hospitals met the definition for ART attrition for an overall ART attrition rate of 26.8 per 100 person-years (95% CI: 23.1–31.2 per 100 PY). Average ART attrition rates for each patient subgroup before vs. after the earthquake are displayed in Table 2. The estimated attrition level at 12 months was 22.8% (95% CI: 19.7–26.2%) at the two hospitals. The 12-month attrition level was higher before vs. after the earthquake (24.4% vs. 20.6%), a marginally significant difference (*p*=0.08). While there were 31 transfers observed in the study population, none were timely transfers which could explain the cases of ART attrition we observed. Figure 3 shows the Kaplan Meier ART attrition curves by facility before vs. after the earthquake.

**Table 2:** Cases and rates of attrition, before and after 2010 earthquake at two hospitals in Haiti

	Attrition	Before		After	
	
	Cases	Rate*	95% CI	Rate*	95% CI
Overall	172	30.0	(24.8–36.2)	23.0	(18.0–29.2)
Site					
Jacmel	54	23.1	(16.8–31.8)	12.5	(7.7–20.4)
Jérémie	118	35.9	(28.3–45.5)	31.4	(23.8–41.4)
Gender					
Male	73	31.1	(23.0–41.9)	24.7	(17.3–35.4)
Female (non-pregnant)	87	28.3	(21.6–36.9)	21.5	(15.3–30.3)
Female (pregnant)	12	37.1	(19.3–71.3)	23.7	(7.6–73.4)
Age group					
<25	16	29.0	(16.0–52.3)	19.1	(7.9–45.8)
25–44	107	30.1	(23.7–38.3)	22.5	(16.5–30.7)
≥45	49	30.0	(20.7–43.5)	25.2	(16.4–38.6)
Commune of residence					
Same as hospital	70	18.9	(14.1–25.4)	16.4	(11.1–24.0)
Adjacent to hospital	47	37.9	(26.7–54.0)	25.9	(15.8–42.2)
Non-adjacent	54	77.0	(53.8–110.1)	36.4	(24.4–54.3)
Missing	1	368.9	(52.0–2619.1)	0.0	
BMI					
<18.5	46	27.5	(19.1–39.6)	20.4	(12.7–32.8)
18.5+	108	27.5	(21.4–35.4)	26.1	(19.6–34.7)
Missing	18	60.0	(36.7–97.9)	8.4	(2.1–33.7)
Baseline CD4					
<100	25	17.8	(10.9–29.1)	17.6	(9.1–33.8)
100–249	61	26.6	(19.1–37.0)	25.9	(17.6–38.1)
250+	45	23.0	(15.3–34.6)	23.4	(15.4–35.5)
Missing	41	99.1	(70.1–140.1)	21.6	(11.2–41.5)
ART regimen					
AZT-3TC-EFV	83	29.6	(22.3–39.3)	21.3	(15.3–29.6)
AZT-3TC-NVP	76	32.7	(24.8–43.2)	25.2	(17.2–37.0)
Other	13	20.6	(10.3–41.2)	25.4	(10.6–61.1)
WHO stage					
Stage I or II	112	27.9	(21.9–35.7)	26.4	(19.9–35.1)
Stage III or IV	55	32.9	(24.0–45.3)	17.3	(10.8–27.9)
Missing	5	42.6	(16.0–113.6)	13.2	(1.9–93.6)
Any WHO stage IV symptom					
No	139	29.2	(23.7–36.0)	24.3	(18.5–32.0)
Yes	33	34.1	(21.5–54.1)	19.3	(11.6–32.0)
TB (treatment, prophylaxis, suspicion)					
No	156	30.7	(25.1–37.5)	24.3	(18.9–31.3)
Yes	16	24.5	(13.2–45.6)	14.8	(6.6–32.9)
Pre-ART duration					
<1 month	90	46.0	(35.6–59.5)	28.4	(20.1–40.1)
1–6 months	41	21.6	(14.3–32.5)	20.1	(12.6–31.9)
>6 months	41	20.6	(14.0–30.6)	18.9	(11.6–30.8)
Counseling sessions					
None	108	31.8	(25.1–40.1)	25.4	(18.5–34.9)
1	55	28.8	(20.6–40.3)	21.2	(13.9–32.6)
2+	9	13.0	(3.3–52.1)	18.1	(8.6–37.9)

* Rate per 100 person years; ART, antiretroviral therapy; AZT, zidovudine; BMI, body mass index; EFV, efavirenz; NVP, nevirapine; TB, tuberculosis; WHO, World Health Organization; 3TC, lamivudine.

In the adjusted time-to-event analysis of ART attrition at the two hospitals (with adjustment for the measured factors shown in Fig. 2), ART attrition declined significantly following the earthquake in Jacmel (HR=0.51 for after vs. before earthquake comparison; *p*=0.03) (see Table 3). However, the decline was more modest over time and lacked statistical significance in Jérémie (HR=0.81 for after vs. before earthquake comparison; *p*=0.29). Compared to patients in Jérémie, patients in Jacmel had a 45% lower risk of attrition before the earthquake (HR=0.55; *p*=0.01) and a 65% lower risk after the earthquake (HR=0.35; *p*=0.001), after adjustment for other factors. The interaction between site and earthquake timing was not significant (*p*=0.20). Apart from hospital site and temporal factors, location of patient residence was strongly associated with ART attrition risk. Patients living outside the commune where the hospital was located had a markedly elevated attrition risk compared to patients living in the same commune as the hospital (HR=2.29; *p*<0.001), after controlling for all other factors. Results for the adjusted analysis were consistent when using other empirical ways of categorizing baseline CD4 and BMI covariates (results not shown).

**Table 3:** Factors associated with ART attrition risk at two hospitals in Haiti

	Hazard ratio	95% CI	*p*-value
Site			
Jacmel (post-quake vs. pre-quake)^a^	0.51	(0.28–0.93)	0.03
Jérémie (post-quake vs. pre-quake)	0.81	(0.55–1.20)	0.29
Jacmel vs. Jérémie (pre-quake)^b^	0.55	(0.35–0.86)	0.01
Jacmel vs. Jérémie (post-quake)^c^	0.35	(0.19–0.65)	0.001
Gender (male=reference)			
Female (non-pregnant)	0.77	(0.50–1.19)	0.24
Female (pregnant)	0.91	(0.44–1.88)	0.79
Age (10 year greater)	1.07	(0.92–1.25)	0.39
Proximity (same commune=reference)^c^			
Adjacent commune	2.25	(1.50–3.39)	<0.001
Non-adjacent commune	2.29	(1.54–3.38)	<0.001
BMI (<18.5=reference)			
>18.5	1.13	(0.76–1.66)	0.55
Baseline CD4 (<100=reference)			
100–200	1.44	(0.81–2.56)	0.20
200+	1.26	(0.65–2.41)	0.47
ART regimen (AZT-3TC-EFV=reference)			
AZT-3TC-NVP	1.36	(0.85–2.18)	0.20
Other regimen	1.17	(0.61–2.23)	0.63
WHO Stage (stage I or II=reference)			
Stage III or IV	1.05	(0.74–1.50)	0.78
TB status (No suspicion, prophylaxis or diagnosis=reference)			
Yes	0.84	(0.48–1.46)	0.54
Any stage IV symptom (No=reference)			
Yes	1.03	(0.69–1.52)	0.90
Pre-ART duration (30 day increase)	0.99	(0.97–1.00)	0.07
Counseling sessions prior to ART start (None=reference)			
1 session	0.92	(0.65–1.30)	0.64
2+ sessions	0.49	(0.24–1.00)	0.05

Jacmel is the site closest to the earthquake epicenter while Jérémie is the site farther from the earthquake epicenter.

ART, antiretroviral therapy; BMI, body mass index; TB, tuberculosis; WHO, World Health Organization; ART regimen: AZT, zidovudine; 3TC, lamivudine; EFV, efavirenz; NVP, nevirapine.

a *p*≤0.05,

b *p*≤0.01,

c *p*≤0.001.

**Fig. 4:**
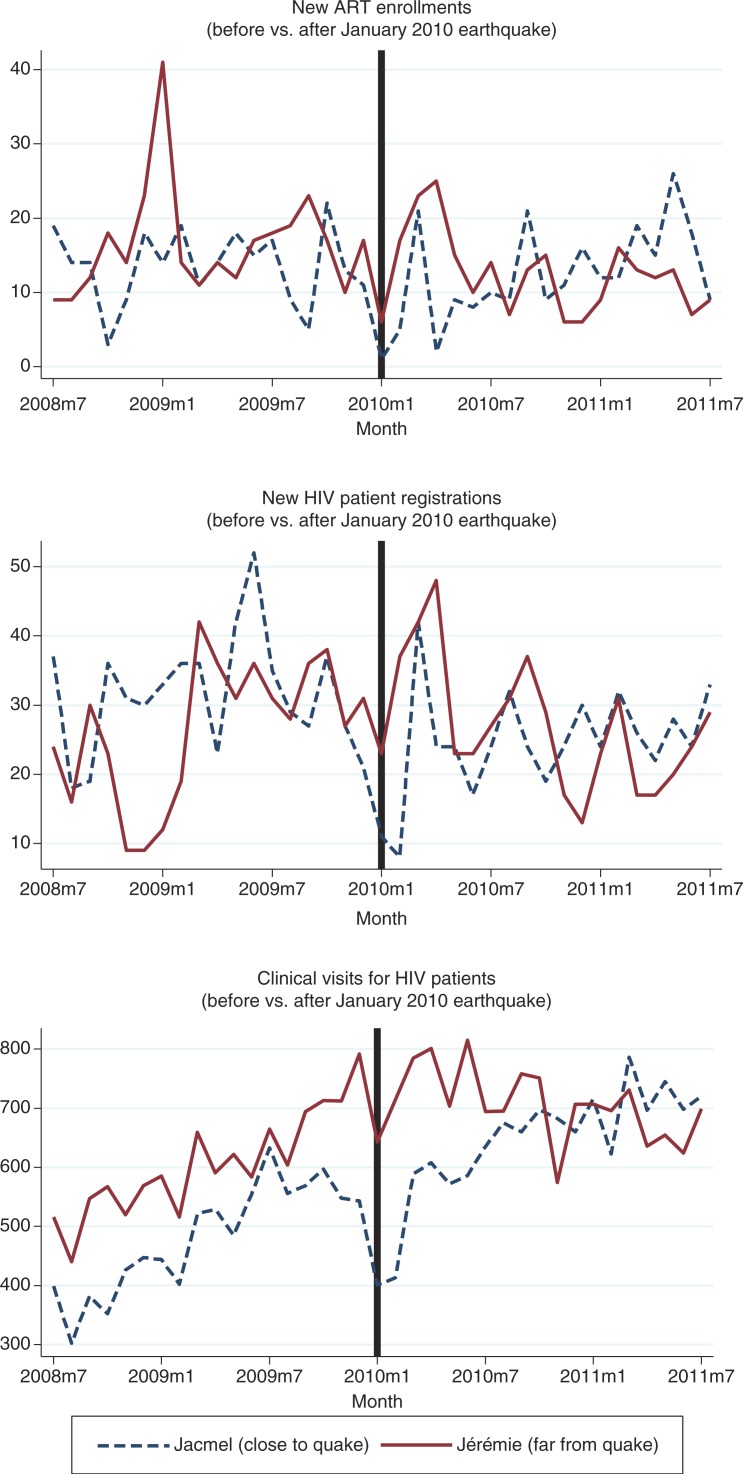
Volume of HIV related services, before and after 2010 earthquake at two hospitals in Haiti.

The volume of HIV-related services provided in Jacmel, the site closest to the quake, dropped steeply in the 2 months immediately following the earthquake, but then generally rebounded within the following months (Fig. 4). There was no significant difference in the average monthly number of new ART enrollments at either site before vs. after the earthquake. Jacmel showed 7.0 fewer new HIV registrations per month on average after the earthquake (*p*=0.05), while Jérémie showed no evidence of difference. In terms of the total number of HIV clinic visits per month, both sites showed significantly more visits per month on average after the earthquake (79.5 more visits per month for Jacmel, *p*=0.05; 89.2 more visits per month for Jérémie, *p*=0.01).

## Discussion

In Jacmel, the site closest to the earthquake, ART attrition improved following the earthquake after adjustment for patient clinical characteristics. Furthermore, adjusted ART attrition was significantly lower in Jacmel than in Jérémie both before and after the earthquake. At both sites, but especially in Jacmel, the number of new ART enrollments, new HIV patient registrations, and number of HIV clinical visits dropped dramatically in January 2010 immediately after the earthquake, but rebounded strongly thereafter. There were no prominent changes to the national ART program model during the 37-month study period, other than a change in 2010 in the national ART treatment guidelines which allowed for expanded use of the first-line regimen of zidovudine–lamivudine–efavirenz (AZT+3TC+EFV) among women of reproductive age. Given the extensive earthquake-related damage and disruption experienced in Jacmel, our findings of a more favorable trajectory of ART attrition in Jacmel and the less favorable trajectory in Jérémie after the earthquake were somewhat surprising.

There are several possible explanations for the patterns we observed. In Jacmel after the earthquake, the quality of ART services could have improved, the site could have restricted ART enrollment to those who were likely to succeed on therapy, or patients could have changed in other ways such that they were more likely to remain in care and on treatment. Our findings support the first explanation. None of the key Haitian HIV care providers were killed, injured, or left the site as a result of the earthquake. Rather, there was an influx of materials and human resources to the site from non-governmental organizations such as the WHO, the United Nations Population Fund (UNFPA), Médecins Sans Frontiers, the Canadian Red Cross, and others. Near-term aid was focused on provision of surgery and trauma care services, stabilization of inpatient services, and restoration of outpatient services, including HIV care and treatment. Over the longer term, the enhanced external aid focused on strengthening health programs. The total number of HIV clinic visits steadily increased, and new ART enrollees were more likely to receive pre-ART counseling sessions in Jacmel. We found no evidence that new ART enrollments were restricted after the earthquake in such a way as to artificially boost ART retention. Patients who survived the life-threatening trauma of the earthquake and later enrolled on ART may have had a tendency to more highly prioritize health and health care, but this type of change is unobservable in our data. Our finding of minimal and highly transitory disruption to ART continuity in Jacmel is consistent with findings from the Walldorf et al. study (11). The favorable trajectory of ART attrition in Jacmel represents an impressive accomplishment.

In contrast, in Jérémie, the site farther from the quake, there was no new influx of external resources to the hospital after the earthquake. The population in and around Jérémie is disproportionately disadvantaged; in the surrounding Grand-Anse Department, fully half of the population is estimated to fall in the lowest quintile of wealth for Haiti (compared to 31% in the South East Department, where Jacmel is located) (23). Outlying communities served by the hospital in Jérémie can be particularly remote and difficult to access, with high transportation costs. These socioeconomic realities may be the most important explanation for the less favorable pattern of ART attrition in Jérémie.

Our case study demonstrated disparities in ART program outcomes between the two facilities, despite adjustment for many socio-demographic and clinical characteristics. This type of disparity is consistent with findings of cross-sectional study of ART attrition among patients enrolled on ART in the year following the earthquake, which was carried out by Haiti's Ministry of Health National AIDS Program (PNLS) (24). This study found that attrition 12 months after ART enrollment varied from 12.8 to 47.8% across facilities with 50 or more patients. Further research is warranted to understand the role of patient and facility characteristics in explaining disparities in ART retention in Haiti.

Our case study contributes evidence that continuity of ART services is possible in settings of humanitarian crisis. As of 2006, the United Nations estimated 7–10% of people with HIV around the globe, representing over 1.7 million people, were affected by complex humanitarian emergencies, resulting from conflict, natural disasters or displacement (25, 26). Several reports have documented the negative effects of such emergencies on health services in general, as well as HIV care and treatment programs specifically. For example, an armed conflict which displaced more than 1 million people in Côte d'Ivoire was associated with a drop of up to 88% in the number of health care workers working actively serving within health facilities within conflict zones (27). In Kenya, post-election violence in 2008 was associated with a 70% greater odds of treatment interruption of >2 days for patients on ART at one urban hospital in Nairobi (28). Massive floods in Mozambique in 2008 led to population displacement, closures of some hospitals and clinics, disruption in pharmacy supplies, outbreaks of water-borne diseases, and other threats to health which negatively impacted continuity of ART services (29).

In contrast, our case study of ART services in Haiti following the 2010 earthquake is a counter-example demonstrating ART program continuity during an emergency situation. Other examples exist. An ART program serving internally displaced people in Northern Uganda reported higher ART adherence rates than those found in other parts of the country where conflict was absent (30). The program credited its successes to a care model which included active follow-up of patients who failed to present for care, extensive adherence counseling, mobile clinic services, and operation of a hotline to guide patients in care-seeking during periods of instability. A non-governmental organization in Mozambique documented success in tracking and sending outreach teams to facilitate a return to care for 60 ART patients who were initially lost to follow up after the 2008 flooding (29). In Haiti, the GHESKIO clinic had a well-established model of ART care involving contingency for times where political protests and unrest in Port-au-Prince made it unsafe for patients to travel to the clinic. GHESKIO activated this contingency plan, enabling patients to obtain an extra 2-week supply of ART medications from pre-designated emergency depots distributed throughout the city, immediately after the earthquake (31). Together these positive examples demonstrate that a close understanding of patients’ complex needs, of potential barriers to accessing care, and of effective contingency measures can each help to mitigate the effects of natural disaster or other humanitarian emergencies on continuity of services for ART patients. The effects of any disaster or emergency on continuity of ART care will depend on the unique combination of baseline conditions, extent of the impact, and level of support mobilized to respond.

A strength of our case study was the availability of detailed person-level clinical data enabling adjustment for many of the factors that might have otherwise biased an analysis of ART attrition before vs. after the earthquake. A limitation is that the study included only two hospitals, and did not include any facilities from areas most violently affected by the earthquake. Despite this limitation, this case study offers a contribution to the literature on HIV service delivery in the context of natural disasters.

## Conclusion

The public health challenges presented by Haiti's devastating 2010 earthquake and subsequent cholera outbreak were profound. Despite this, we found no strong evidence of association between earthquake intensity and ART attrition patterns, based on a longitudinal comparison of two hospitals differently affected by the earthquake. That ART attrition improved at Hôpital St. Michel in Jacmel following the 2010 earthquake was an important accomplishment, reflecting the resilience of hospital staff and patients and the role of external aid in stabilizing the national ART program in the post-earthquake period.
